# Epidemic Trend and Molecular Evolution of HV Family in the Main Hantavirus Epidemic Areas From 2004 to 2016, in P.R. China

**DOI:** 10.3389/fcimb.2020.584814

**Published:** 2021-02-03

**Authors:** Qiuwei Wang, Ming Yue, Pingping Yao, Changqiang Zhu, Lele Ai, Dan Hu, Bin Zhang, Zhangnv Yang, Xiaohong Yang, Fan Luo, Chunhui Wang, Wei Hou, Weilong Tan

**Affiliations:** ^1^ Department of Infectious Disease Prevention and Control, Eastern Theater Command Centers for Disease Control and Prevention, Nanjing, China; ^2^ Department of Infectious Diseases, The First Affiliated Hospital of Nanjing Medical University, Nanjing, China; ^3^ Department of Microbiological Test, Zhejiang Provincial Center For Disease Control and Prevention, Hangzhou, China; ^4^ State Key Laboratory of Virology/Institute of Medical Virology, School of Basic Medical Sciences, Wuhan University, Wuhan, China

**Keywords:** hantavirus, epidemical tendency, incidence rate, genetic variation, molecular evolution

## Abstract

Hemorrhagic fever with renal syndrome (HFRS) is caused by hantavirus (HV) infection, and is prevalent across Europe and Asia (mainly China). The genetic variation and wide host range of the HV family may lead to vaccine failure. In this study, we analyzed the gene sequences of HV isolated from different regions of China in order to trace the molecular evolution of HV and the epidemiological trends of HFRS. A total of 16,6975 HFRS cases and 1,689 HFRS-related deaths were reported from 2004 to 2016, with the average annual incidence rate of 0.9674 per 100,000, 0.0098 per 100,000 mortality rate, and case fatality rate 0.99%. The highest number of cases were detected in 2004 (25,041), and after decreasing to the lowest numbers (8,745) in 2009, showed an incline from 2010. The incidence of HFRS is the highest in spring and winter, and three times as many men are affected as women. In addition, farmers account for the largest proportion of all cases. The main hosts of HV are *Rattus norvegicus* and *Apodemus agrarius*, and the SEOV strain is mainly found in *R. norvegicus* and *Niviventer confucianus*. Phylogenetic analysis showed that at least 10 HTNV subtypes and 6 SEOV subtypes are endemic to China. We found that the clustering pattern of M genome segments was different from that of the S segments, indicating the possibility of gene recombination across HV strains. The recent increase in the incidence of HFRS may be related to climatic factors, such as temperature, relative humidity and hours of sunshine, as well as biological factors like rodent density, virus load in rodents and genetic variation. The scope of vaccine application should be continuously expanded, and surveillance measures and prevention and control strategies should be improved to reduce HFRS infection in China.

## Introduction

Hemorrhagic fever with renal syndrome (HFRS) is a zoonotic disease caused by HV, and is characterized by fever, renal failure and hemorrhagic symptoms. It is prevalent across Europe and Asia, and China accounts for 90% of all global cases (Mc and Hart, 2000; Zhang et al., 2010a). HFRS is designated as a legal class B infectious disease in China, which warrants prevention and control in strict accordance with the relevant regulations (Chen et al., 1986; Schmaljohn and Hjelle, 1997). After the first occurrence of HFRS in mainland China in the 1980s, the incidence rate fluctuated in the following decade but showed an overall downward trend. During the 1990s, 40,000 to 60,000 cases were reported each year (Bai et al., 2019), and declined thereafter due to implementation of measures like effective rodent control, environmental management and vaccination, resulting in the lowest incidence in 2009 (Zuo et al., 2011). In recent years however, the incidence of HFRS has increased and new cases have been reported in 31 provinces of China.

Hantavirus (HV) is a single-stranded negative-strand RNA virus that belongs to the order *Bunyavirales*, family *Hantaviridae* and genus *Orthohantavirus*. It is the main causative agent of HFRS and hantavirus pulmonary syndrome (HPS), and is transmitted by small mammals such as rodents, insectivorous animals and bats (Schmaljohn and Dalrymple, 1983; Plyusnin, 2002; Jonsson et al., 2010). The HV genome consists of large (L), medium (M) and small (S) segments, which respectively encode viral RNA-dependent RNA polymerase, viral glycoprotein G1 and G2, and viral nucleocapsid protein (NP) (Plyusnin et al., 1996). Twenty-two HVs have been identified so far, of which 11 are found in China. HTNV and SEOV are the main genotypes responsible for HFRS in China, and are respectively transmitted by *Apodemus agrarius* and *Rattus norvegicus* (Zou et al., 2008a; Zhang et al., 2011; Liu et al., 2012; Li et al., 2019). In addition, other HVs like the *Thottapalayam virus* (TPMV), *Gou virus* (GOUV), *Longquan virus* (LQUV), *Da Bie Shan virus* (DBSV), and *Amur-Soochong virus* (ASV) have also been detected in insectivorous animals, bats and other rodents in China. Nevertheless, their disease causing potential in humans remains unclear (Wang et al., 2000; Zhang et al., 2010a; Guo et al., 2011; Lin et al., 2012a). Phylogenetic analyses show that each HV is transmitted by a specific host, and that viruses have co-evolved with their respective hosts (Guo et al., 2013).

Climatic conditions affect the abundance of HV hosts, and therefore disease transmission dynamics. The infection rate of HV gradually increases with warmer temperatures (Prist et al., 2017). In recent years, numerous HFRS cases have been reported in the Fujian, Zhejiang and Shaanxi provinces of China. According to the Chinese Center for Disease Control and Prevention, the number of cases in Zhejiang, Jiangsu and other provinces have shown an upward trend since 2011 (Liu et al., 2016; Zou et al., 2016; Wu H. et al., 2018; Xiang et al., 2018). In this study, we analyzed the gene sequences of HV isolated from different regions of China and other countries, and traced their evolution through genetic analysis. In addition, the epidemiological characteristics and trends of HFRS were also investigated to provide a theoretical basis for its effective prevention and control.

## Materials and Methods

### Data Extraction

Since 2004, HFRS cases from all across China have been directly reported to the CDC. Demographic (age, sex, and occupation) and epidemiological (regional distribution, time trend, and population distribution) data of all clinically confirmed HFRS cases reported between 2004 and 2016 were extracted from the legal infectious disease reporting system of the Chinese CDC (http://www.phsciencedata.cn/Share/). The cases had been diagnosed on the basis of the Chinese Diagnostic Criteria of Epidemic Hemorrhagic Fever (GB15996-1995 and ws278-2008). The annual incidence rates were used to analyze the time trend of HFRS, and the seasonal trends were evaluated in provinces with high prevalence. The distribution of HV genotypes was extracted from existing literature and reports published in the PubMed and Chinese National Knowledge Infrastructure (CNKI) databases. The complete genomic sequence of the M segment of 73 HV strains and S segment of 86 HV strains were acquired from National Center Biotechnology Information (NCBI).

### Data Analysis

SPSS 23.0 software was used to analyze the demographic data, and different groups were compared by the chi-square test. *P*<0.05 was considered statistically significant. Epi info software was used to analyze the regional distribution of HFRS from 2004–2016. The genetic analysis of HVs in the different regions was performed with DNAstar software, and a phylogenetic tree was constructed using the MEGA-X 6.0 software. Genetic re-assortment analysis was performed using Simplot software. All sequences were extracted from GenBank (**Table S1**).

## Results

### Epidemic Overview of HFRS in China

A total of 166,975 HFRS cases and 1,689 HFRS-related deaths were reported in 31 provinces between 2004 and 2016. The average annual incidence was 0.9674/100,000, average annual mortality was 0.0098/100,000, and the average annual fatality rate was 0.9924% (**Table 1**). During this period, most cases (25,041) occurred in 2004 and the fewest in 2009 (8,745 cases). The highest mortality rate of 0.02/100,000 was recorded in 2005, and the highest case-fatality rate was 1.31% in 2007.

**Table 1:** Overview of hemorrhagic fever with renal syndrome (HFRS) incidence in China from 2004 to 2016.

Years	Number of cases	Deaths	Incidence (/100,000)	Mortality rate (/100,000)	Case fatality rate (%)
2004	25,041	254	1.9264	0.0195	1.0143
2005	20,877	271	1.6061	0.0208	1.2981
2006	15,098	173	1.1547	0.0132	1.1458
2007	11,063	145	0.8416	0.011	1.3107
2008	9,039	103	0.6841	0.0078	1.1395
2009	8,745	104	0.6585	0.0078	1.1893
2010	9,526	118	0.7137	0.0088	1.2387
2011	10,779	119	0.8039	0.0089	1.1040
2012	13,308	104	0.9877	0.0077	0.7815
2013	12,810	109	0.9461	0.008	0.8509
2014	11,522	79	0.8502	0.0058	0.6856
2015	10,314	62	0.7570	0.0046	0.6011
2016	8,853	48	0.6458	0.0035	0.5422

### Epidemic Characteristics of Hemorrhagic Fever With Renal Syndrome in China

#### Regional Distribution

**Figure 1:**
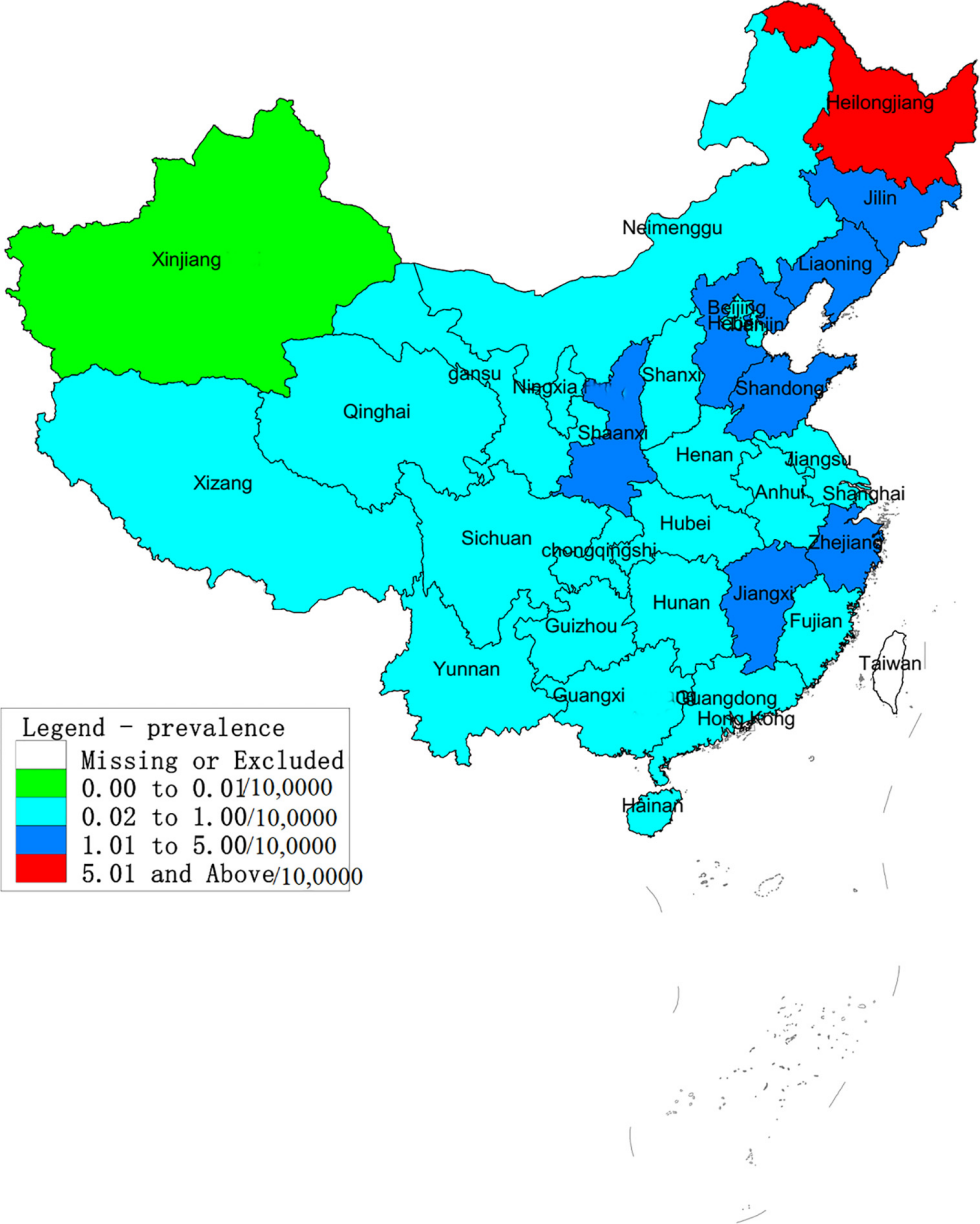
Annual average incidence of hemorrhagic fever with renal syndrome (HFRS) in provinces across China from 2004 to 2016. According to the average annual incidence rate, the regions in China were divided into four categories: low epidemic – 0–0.01/10,0000; moderate endemic - 0.01–1/10,0000; high-endemic – 1–5/10,0000; very highly endemic > 5/10,0000. Heilongjiang was classified as a highly endemic area and Shaanxi, Jilin, Liaoning, Shandong, Hebei, Jiangxi and Zhejiang. Xinjiang is a low-endemic area. All other areas are considered moderately endemic.

The regions with the highest number of cases were Heilongjiang, Shaanxi, Liaoning, Shandong, Jilin and Hebei, followed by Hunan, Zhejiang, Jiangxi and Jiangsu. A total of 142,470 cases were reported in these provinces, and accounted for 85.32% of all cases. The average annual incidence of HFRS across the different provinces ranged from 0.005/100,000 to 6.027/100,000, and showed significant differences. As shown in **Figure 1**, Heilongjiang had the highest incidence of HFRS, and can be considered highly endemic. Shaanxi, Jilin, Liaoning, Shandong, Hebei, Jiangxi and Zhejiang were moderately endemic, and Xinjiang was designated as a low epidemic area.

#### Annual and Seasonal Distribution

**Figure 2:**
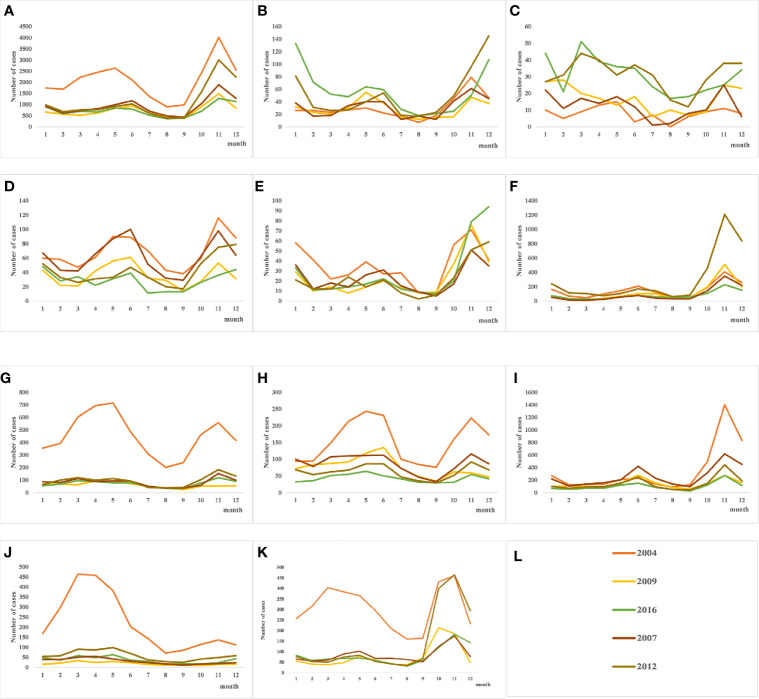
Quarterly distribution of hantavirus (HV) cases in several provinces with severe epidemics. From 2004 to 2016, the incidence of HFRS in each province showed an overall downward trend. **(A)** The seasonal trends of hemorrhagic fever with renal syndrome (HFRS) incidence throughout the country shows summer and autumn/winter peaks, and the autumn peak is higher. **(B–E)** Bimodal seasonal peaks in Jiangxi, Fujian, Zhejiang and Jiangsu provinces in Southeast China. **(F–K)** HFRS initially occurred in colder months in the Shaanxi, Liaoning, Jilin, Heilongjiang, Hebei, and Shandong provinces in North China, but the recent trend is bimodal. **(L)** shows the legend used by **(A–K)**.

As shown in **Table 1**, the incidence of HFRS in China gradually decreased from 2004 to 2009, and the highest annual incidence (1.9264/1,000,000) was observed in 2004 and the lowest (0.6458/100,000) in 2016. Similar trends were observed in the individual provinces as well. The number of cases in Fujian increased steadily since 2012, whereas Shaanxi province showed a sudden spike in 2012. In addition, the incidence of HFRS also showed a seasonal trend, with bimodal change from May to July and from November to January. The peak incidence was in June during summer and in November during autumn and winter seasons, although the overall incidence was higher in the colder months (**Figure 2**). Finally, the prevalence of HFRS has steadily shifted from a mixed epidemic area dominated by *A. agrarius* to that dominated by *R. norvegicus*.

#### Demographic Characteristics of HFRS Cases

**Figure 3:**
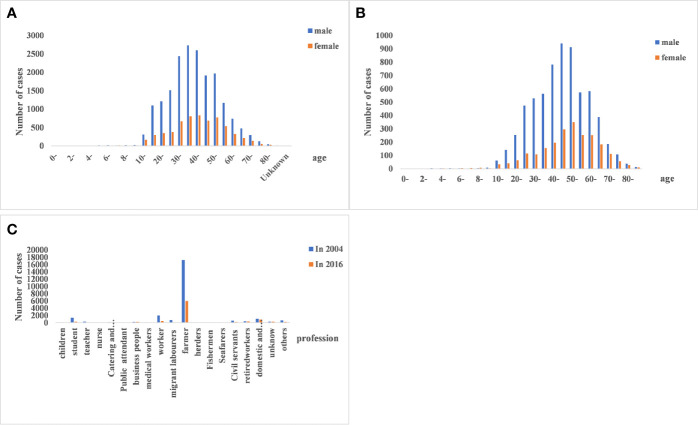
Age, gender, and occupational distribution of hemorrhagic fever with renal syndrome (HFRS) cases. **(A, B)** HFRS cases in the different age- and gender groups between 2004 and 2016. The 15–64 age group and males were predominantly affected. **(C)** Occupation of HFRS patients. Farmers are the main affected population.

The demographic characteristics of HFRS cases in 2004 and 2016 were analyzed. The male to female ratio was 3:1 (*P*<0.01; **Table 2**), and 88.71% of the patients were between 15 and 64 years of age (*P*<0.01 compared to the other age groups; **Table 3**). Furthermore, farmers accounted for 67.85% of the total cases (**Figure 3**).

**Table 2:** Hemorrhagic fever with renal syndrome (HFRS) incidence based on gender.

Sex	Case	Healthy	Chi-Square	*P^a^*
male	6,574	708,143,426	1,881.374	<0.01
female	2,279	674,557,721		

P^a^ the difference of sex.

**Table 3:** Hemorrhagic fever with renal syndrome (HFRS) incidence in different age groups.

Age	Case	Healthy	Chi-Square	*P^b^*
<15	156	222,393,364	1,460.742	<0.01
15-64	7,576	998,083,824		
>=65	1,121	119,233,959		

P^b^ the difference of age.

### Genetic Variation Characteristics and Phylogenetic Analysis of Hantavirus (HV)

#### Genetic Characteristics of HV

**Figure 4:**
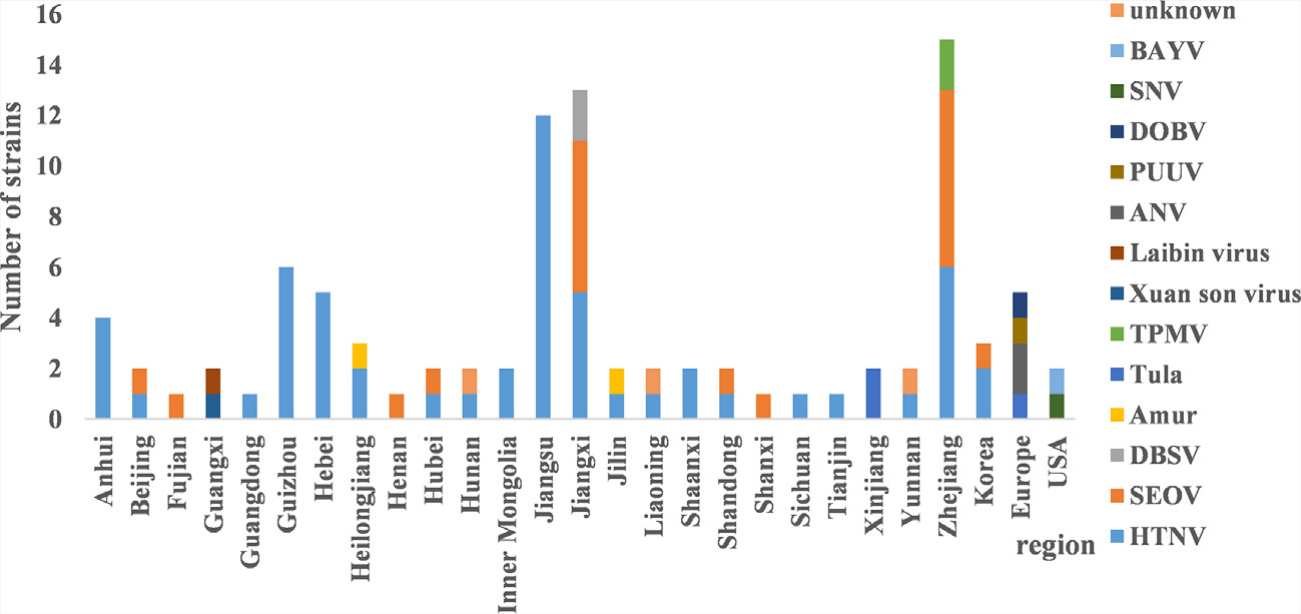
The distribution of hantavirus (HV) genotypes across different regions. HTNV and SEOV were predominant in most provinces. There were three distinct genotypes in Jiangxi and Zhejiang respectively. The main genotypes in Guangxi and Xinjiang were Xuan son, LAIBIN, and TULA.

**Figure 5:**
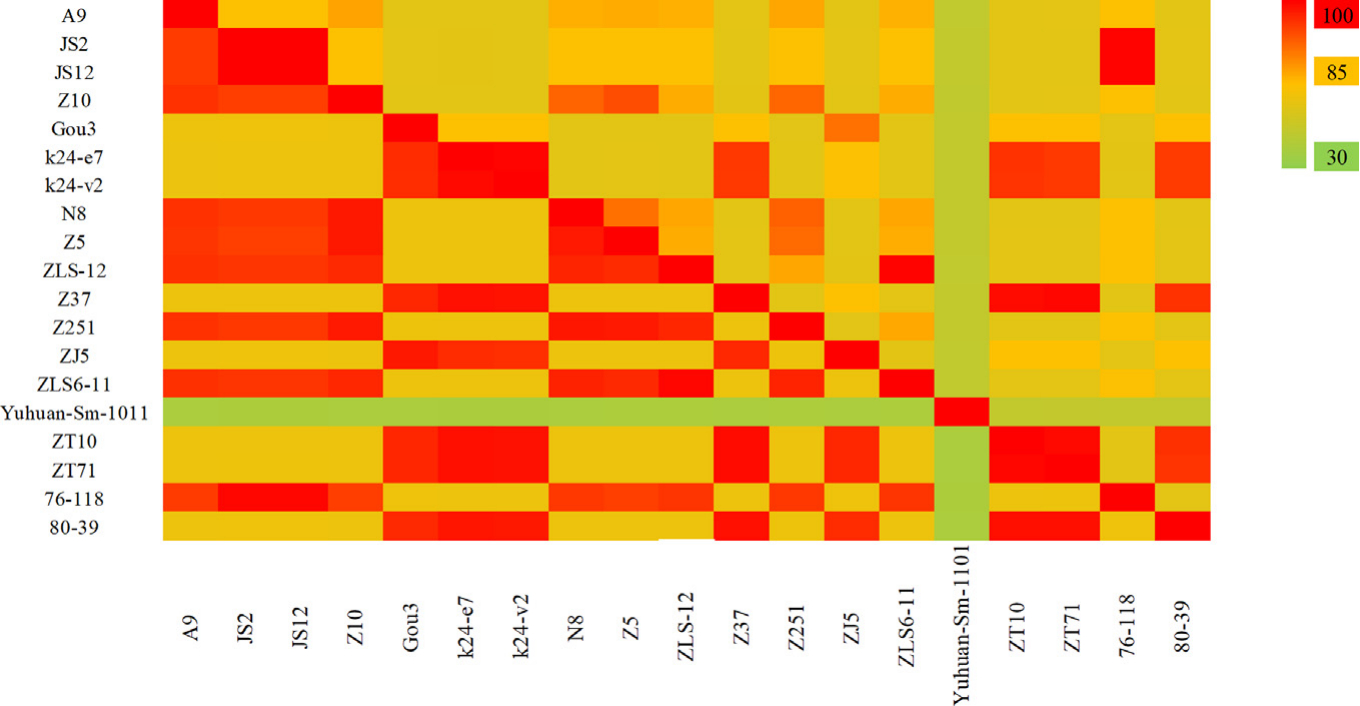
The hantavirus (HV) M segment sequence homology analysis in China. The upper triangle region represents the nucleotide sequence homology and the lower triangle region indicates amino acid homology. The nucleotide and amino acid homology between HTNV strains in Jiangsu Province were 84.6% and 95.4%. The nucleotide and amino acid homology of HTNV strains in Zhejiang Province were 86.5%–99.8% and 96.8%–99.6%, and of SEOV were 83.8%–99.7% and 96.4%–99.5% respectively. The Yuhuan-Sm-1011 strain is a new genotype distinct from HTNV and SEOV.

HV genome sequences were downloaded from GenBank, and a phylogenetic tree was constructed on the basis of homology analysis. The virus strains used for genetic analysis are listed in **Table S1**. The genotypic distribution across different provinces is shown in **Figure 4**. According to the nucleotide and amino acid sequences analysis of different segments of HV, all the strains isolated in the Jiangsu Province were HTNV, which had 100% nucleotide homology with the JS1-12 strain and 99.8% homology with the prototype of HTNV76-118. The A9 strain showed 16.1%–18.7% nucleotide divergence from the other strains in Jiangsu Province. Furthermore, the A9 strain had 83.8% nucleotide homology and 95.6% amino acid sequence homology with HTNV76-118 strain. The M segment analysis of HV obtained from Zhejiang Province showed 86.5%–99.8% and 96.8%–99.6% nucleotide and amino acid homology respectively with the local HTNV strains. However, the nucleotide divergence between these strains and HTNV76-118 was 17.3%–18.5%. The nucleotide and amino acid homology among the SEOV strains in Zhejiang Province was 83.8%–99.7% and 96.4%–99.5% respectively, and the proportion of homologous nucleotide and amino acid sequences between these strains and the international standard strain SEOV80-39 were 95.4%–96.3% and 96.6%–98.9% respectively. Interestingly, the SEOV virus strains Gou3 and ZJ5 had 84.2% nucleotide homology with SEOV80-39, although 96.6%–96.8% of the amino acid sequences were homologous. In addition, the TPMV virus found in the shrews in Zhejiang Province showed only 62%–62.9% nucleotide and 61.1%–62.2% amino acid homology with the southeast coast and international standard strains, indicating significant genetic differences. The results are summarized in **Figure 5**.

**Figure 6:**
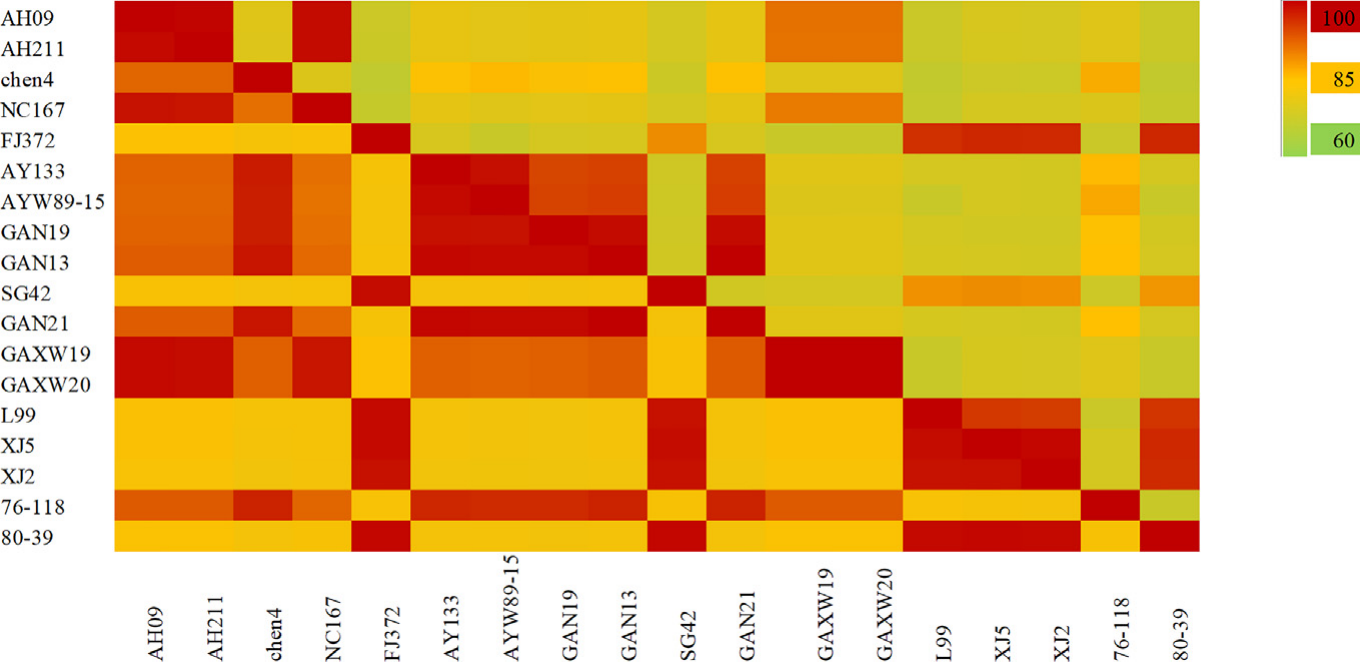
The hantavirus (HV) S segment sequence homology analysis in China. The upper triangle region represents the nucleotide sequence homology and the lower triangle region indicates amino acid homology. There was 77%–99% nucleotide homology and 91%–99% amino acid homology between HTNV strains in Anhui province. The nucleotide and amino acid homology of HTNV strains in Jiangxi Province were 94.6%–100% and 98.6%–100%, and of SEOV were 88%–99.5% and 98.6%–99.1% respectively.

As shown in **Figure 6**, the S segment analysis indicated that the four strains found in Anhui Province were HTNV. The nucleotide and amino acid sequence homology among the AH09, AH211 and NC167 strains was 99.2%–99.7% and 98.4%–99.3% respectively, and all three were genetically distinct from HTNV76-118, with 76.6%–79.5% nucleotide and 92.1%–93% amino acid homology. The chen4 virus strain was also significantly different from the other three strains with 27% nucleotide divergence, and 86.6% nucleotide and 97.4% amino acid homology with HTNV76-118. The virus strains found in Jiangxi Province included five HTNV, four SEOV and two DBSV strains. The nucleotide homology between the HTNV strains was 94.6%–100% and the amino acid homology was 98.6%–100%, and were distinct from HTNV76-118. The S segment nucleotide homology among the SEOV virus strains L99, XJ2 and XJ5 was 95.3%–99.5%, and the three strains had 95%–97% homology with SEOV80-39 and Fj372/2013. The strain SG42 showed 88.7%–89.1% nucleotide homology with other SEOV virus strains, and only 88.4% with SEOV80-39. However, as seen with the M segment analysis, the amino acid homology between SEOV strains in the Jiangxi Province was 98.6%–99.1%. These results indicate that the nucleotides of this strain can have synonymous mutations. The homologous nucleotide and amino acid sequences between DBSV and HTNV were 75.6%–78.3% and 92.3%–93% respectively. Both showed high nucleotide and amino acid homology of 90.4%–91.2% and 98.4%–99.3% respectively with the virus strain found in Dabieshan in the Anhui Province.

#### Phylogenetic Analysis of HV

**Figure 7:**
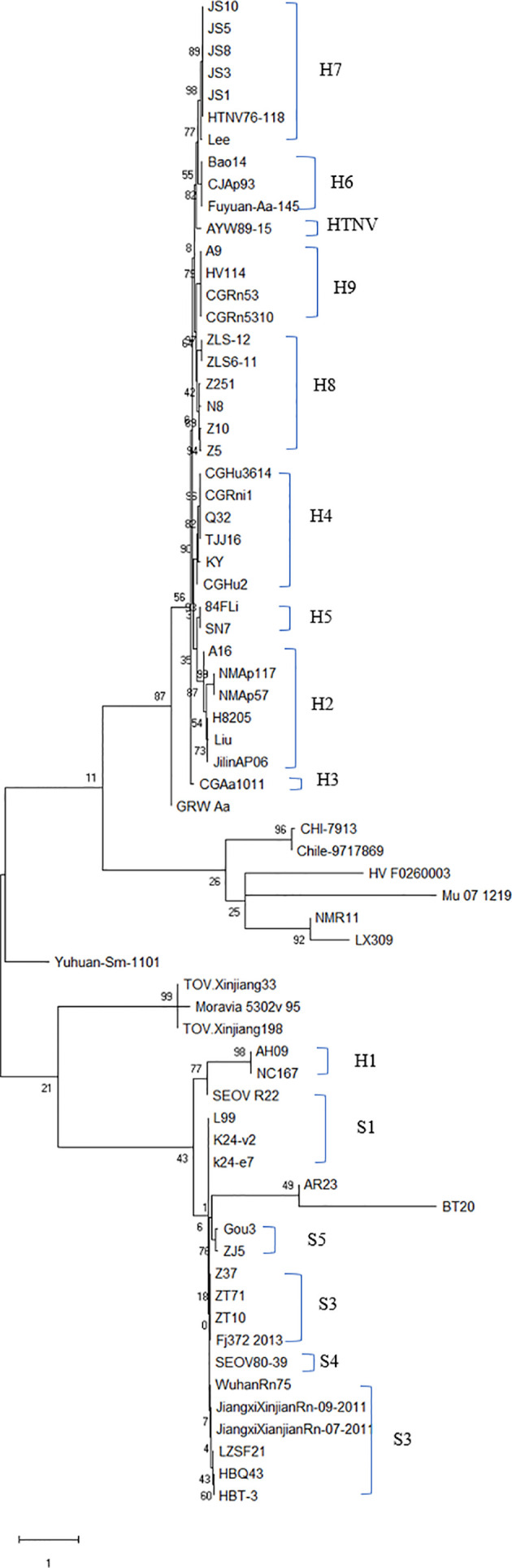
Phylogenetic tree of hantavirus (HV)-based on the open reading frame of M segments sequence. The tree was constructed by the maximum likelihood method (ML) of Mega software. A new HV type was identified if the nucleotide sequence of at least one fragment was >25% different from other known HV sequences, and a new subtype was the result of 5% sequence difference. Accordingly, nine subtypes were identified for HTNV and five for SEOV. Nucleic acid sequence difference < 5% indicated the same subtype. Subtypes H7 and H8 were mainly found in the southeast coastal areas. The GenBank accession numbers of the viruses are shown in **Table S1**.

**Figure 8:**
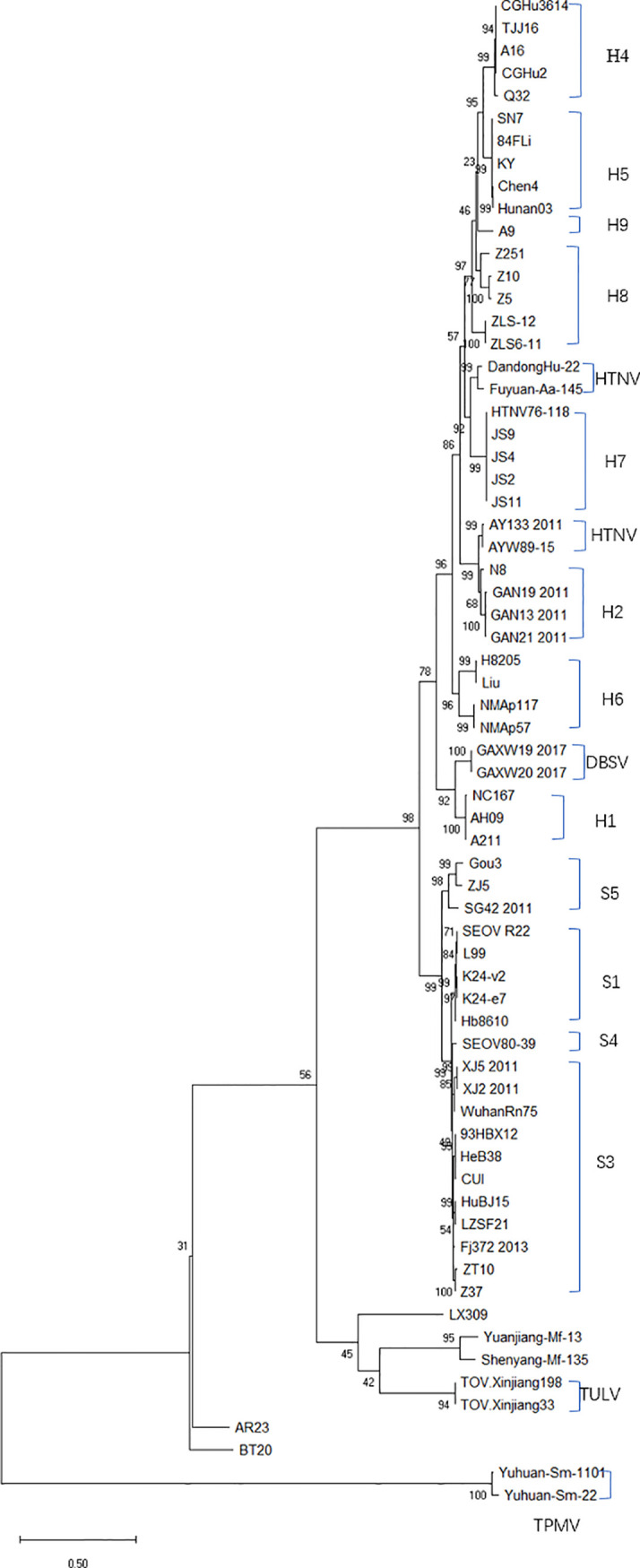
Phylogenetic tree (ML) of HV-based on the open reading frame of S segments sequence. SEOV is widely distributed in China, and the subtypes of SEOV in the southeast coastal areas are mainly S1 and S3. The GenBank accession numbers of the viruses is shown in **Table S1**.

**Figure 9:**
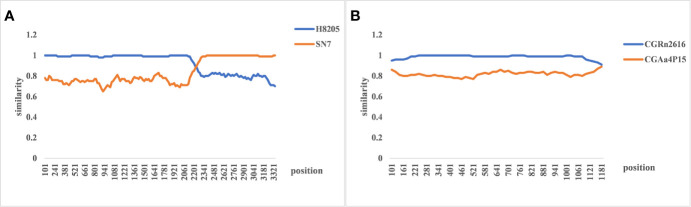
The re-assortment analysis of complete sequences of hemorrhagic fever with renal syndrome (HFRS). **(A)** The M segment re-assortment of A16 virus strain. The assortment of the H8205 and SN7 virus strain. The recombination breakpoint is 2,214–3,408 bp, which indicates that hantavirus (HV) with the same subtype and similar host is more likely to undergo gene recombination. **(B)** The re-assortment analysis of CGRn2616 and CGAa4P15 based on the S segment, indicating no gene assortment.

The evolutionary relationship among the HV types (HTNV, SEOV, TULV, TPMV, and PUUV, and their subtypes) from different regions were next analyzed by constructing phylogenetic trees based on the coding region sequences of M and S segments. The maximum likelihood method similar to the neighbor-joining method was used. We detected a clear regional cluster for HV, along with significantly different types across geographical regions. The distance between the HV strains in the southeast coastal areas was relatively close and the homology was high, indicating a common ancestor. Furthermore, 16.1% divergence between the JS strain in Jiangsu and the A9 strain found earlier indicated recent genetic variations in the HVs in Jiangsu, and the emergence of a new subtype. According to M fragment analysis, the distance between strains A9 and CGRn53 from Guizhou and HV114 from Hubei was relatively close, and the nucleotide homology was 98.9%–99.5%. Regardless of the phylogenetic tree, the SEOV strains from Hebei, Shandong, Jiangxi and Zhejiang provinces are of the S3 subtype, indicating widespread distribution in China. The HTNV strains isolated from Sichuan and Shaanxi belong to the H5 subtype. Furthermore, the HTNV strain from Guizhou was closely related to the strain found in Tianjin, all of which belong to the H4 subtype. The HV strains isolated from Shenyang and Hunan were relatively close, with 85% nucleotide homology. In addition, we detected considerable distance between the Yuhuan-Sm-1101 strains from Zhejiang Province and HV strains from other provinces, and identified a new genotype. The HTNV strains were isolated from rats and voles in different provinces, and SEOV was found in *A. agrarius*, indicating significant host spillover of HV in China (**Figures 7** and **8**). Furthermore, the M segment of A16 virus strain isolated from *A. agrarius* was the recombinant strain, and its primary and secondary parental strains were respectively H8205 isolated from *A. peninsulae* in Heilongjiang and SN7 isolated from *Niviventer confucianus* in Sichuan. However, the CGRn2616 and CGAa4P15 strains could not be assorted based on the S segment (**Figure 9**).

#### Ecology Analysis of HV

HTNV, SEOV, DBSV, AMUR and TULA are highly prevalent in mainland China, especially HTNV and SEOV. The former are mainly distributed in east China and several inland provinces of Guizhou, Yunnan, Shaanxi and Hunan (Zou et al., 2008a), whereas SEOV is present in the central provinces (Liu et al., 2017). DBSV, AMUR, TULA, LAIBIN, and TPMV were detected in one or two provinces (Zhang, 2014), and were mainly isolated from the lung, serum and gastric mucosa of infected patients (Guo et al., 2017). All HVs were always detected in their primary hosts. The potential zoonotic hosts of HTNV are *A. agrarius*, *R. norvegicus*, *R. norvegicus Rerkonhout*, *Microtus fortis Buchner*, *N. confucianus*, *Mus musculus*, *A. peninsulae*, white rat, and *R. nitidus*. SEOV is mainly found in *R. norvegicus*, *M. fortis*, *A. agrarius* and *Suncus murinus* (Lin et al., 2012b; Wu Z et al., 2018) (**Table 4**).

**Table 4:** Regional distribution and host characteristics of hantavirus in China.

VIRUS TYPE	Region	Hosts	Sequence origin	Time
HTNV	Jiangsu, Zhejiang, Anhui, Jiangxi, Heilongjiang, Guizhou, Shandong, Shaanxi, Tianjin, Hubei, Yunnan, Guangdong, Liaoning, Hunan, Jilin	*Apodemus agrarius*, *Rattus norvegicus*, *Rattus norvegicus Rerkonhout*, *Microtus fortis Buchner*, *Human, Niviventer confucianus*, *Mus musculus*, *Apodemus peninsulae*, *White rat*, *Rattus nitidus*	Lung, serum, gastric mucosa in patients with acute HFRS, cell culture stocks, Vero E6 cells	1981–2015 (Li et al., 2020)
SEOV	Zhejiang, Jiangxi, Fujian, Henan, Hubei, Hebei, Shanxi, Beijing	*Rattus norvegicus*, *Microtus fortis*, *Apodemus agrarius*, *Rattus norvegicus*, *Suncus murinus*, *Human*	Serum, gastric mucosa in patients with acute HFRS	1981–1989 (Zhang et al., 2010b; Liu et al., 2012)
DBSV	Jiangxi	*Niviventer confucianus*	lung	2017 (Liu et al., 2018)
AMUR	Heilongjiang, Jilin	*Apodemus peninsulae*	Lung, serum	2012 (Chen et al., 2012), 2007 (Jiang et al., 2007)
TULA	Xinjiang	*Microtus obscurus*	Lung	2016 (Chen et al., 2019)
Xuan son virus	Guangxi	*Hipposideros cineraceus*	lung	2015 (Xu et al., 2019)
LAIBIN	Guangxi	*Taphozous melanopogon*	lung	2012 (Xu et al., 2019)
TPMV	Zhejiang	*shrew*	Lung	2011 (Lin et al., 2014)

## Discussion

The incidence of HFRS declined in China from 2004 to 2009, and showed a slight incline from 2010 to 2016. The expanded anti-HV immunization program for the 16–60 years age group in 2008 significantly decreased the number of HFRS cases in 2009 (Brocato and Hooper, 2019). In addition, greater public awareness, effective rodent control and socio-economic changes have also potentially contributed to the overall decrease in the incidence of HFRS in most provinces. However, an upward trend was observed in Shaanxi, Shandong and Jiangxi provinces since 2010, which can be attributed to climate change that may have altered rodent habitats and population density, the change in HV load caused by host migration, entry of non-immune people into epidemic areas and other factors. Furthermore, the decline in morbidity in recent years may also have slackened the preventive measures and public awareness, resulting in the rebound of HFRS (Jiang et al., 2016).

HFRS occurrence showed a bimodal distribution, with a spring peak from May to July and a winter peak from November to January. The seasonal variations can be attributed to virus type, host reproduction and activity, and other natural and social factors (Zhang et al., 2009; He et al., 2018). For instance, the incidence curve of HFRS transmitted by *A. agrarius* was bimodal as described above, and the peak incidence in the colder months was significantly higher than that in spring. In contrast, the peak HFRS incidence transmitted by *R. norvegicus* is seen in spring (Zhang et al., 2014). Furthermore, our data indicated that HFRS epidemic has shifted from regions dominated by *A. agrarius* to that dominated by *R. norvegicus*. While the dominant HV host in Jiangsu province is *A. agrarius*, Jiangxi and Zhejiang are mixed epidemic areas of *A. agrarius* and *R. norvegicus*. The rapid economic development and environmental changes in recent years has increased contact between humans and rodents, which is highly conducive to the spread of HV (Su et al., 2019). Furthermore, we found that the occupational and gender distribution of HFRS cases in 2016 were consistent with that in 2004. The majority of HFRS cases were male farmers, since they are more likely to work in rodent-infested areas such as barns and fields. In China, the living and working environment of farmers increases exposure to rodent feces, urine and saliva, which further increase the risk of infection in this group (Vapalahti et al., 1999).

Genetic analysis of HVs isolated from rodents, humans and other hosts in different regions of China revealed 10 subtypes of HTNV and at least 6 subtypes of SEOV, which showed distinct geographical aggregation. Previous studies have shown that each HV genotype has a specific host, and the HV genotypes that form distinct regional clusters are significantly different (Wang et al., 2000; Liu et al., 2012). Dabieshan is the watershed between the Yangtze River and the Huaihe River connecting Henan, Hubei and Anhui provinces, and has a high incidence of HFRS. The nucleotide homology among HTNV strains from Dabieshan was 90.4%–99.7%, and were genetically distinct from virus strains from the southeast coastal area. The SEOV strains isolated from Zhejiang belong to different subtypes, and therefore show obvious genetic diversity. In addition, TPMV and TULA virus isolated respectively from Zhejiang and Xinjiang were similar to Moravia/5302v/95 from Europe. Phylogenetic analysis indicated a cluster of HTNV from Shandong, Anhui, Zhejiang and Jiangxi provinces, which may correspond to the migration of *A. agrarius* along the Yangtze River. Since HV is a single-stranded negative-strand RNA virus, it is prone to gene re-assortment and mutation (Zou et al., 2008b; Nikolic et al., 2014). The M segment sequences indicated a gene re-assortment between H8205 and SN7, which further suggested that gene recombination occurs between the different HTNV types. In addition, previous studies have shown genetic assortment between CGRn8316 and CGRn9415 in Guizhou, indicating that HTNV and SEOV may also undergo re-assortment. The phylogenetic tree based on S fragments showed significant genetic distance between the Chen4 strain and three other strains isolated from Anhui Province, with only 77.2%–77.5% nucleotide sequence homology. This also indicates the possibility of a gene mutation in this strain. Most studies show that each HV genotype is associated with a specific rodent host. For instance, HTNV is mainly carried by *A. agrarius* and the primary host of SEOV is *R. norvegicus* (Watson et al., 2014). However, we found that the HTNV strains in Jiangsu Province exist in both animals, and the SEOV in Zhejiang province is also present in voles, indicating host spillover across different genotypes of HV. In addition, genetically diverse HVs were also found in the bats from Guangxi. The genetic distance between ShenyangMf-135 isolated from Shenyang and Yuanjiang-Mf-13 from Hunan was relatively close, indicating that the sequence homology is greater among HVs isolated from the same host. Genetic variations in HVs are the result of host adaptation to environmental changes. However, we mainly analyzed the HV sequences obtained from rodents rather than the other hosts such as bats, shrews etc (Gu et al., 2014). Taken together, the genetic diversity of HV is the result of cross-species transmission, virus-host co-evolution, and host migration (Lin et al., 2012a).

The HFRS cases analyzed in this study were diagnosed on the basis of epidemiological history, clinical manifestations and laboratory tests. Besides, serological diagnosis of HV from different hosts by extracting RNA, performing RT-PCR and sequencing to analyze its epidemic types is essential and accurate. Therefore, the information on HFRS cases and hantavirus genotypes were accurate and reliable. There are a few limitations in thus study that ought to be considered. Since we could not obtain the serological data of the documented cases, it was not possible to analyze the epidemiological characteristics of different HV genotypes. In addition, we did not examine the host species and density in different regions, and therefore cannot draw a conclusion on virus and host co-evolution. Finally, we only analyzed the whole gene sequence of M and S segments, and not the L fragment and partial sequences of virus strains, which limits our inferences. Therefore, future studies should expand on the related gene sequences from different regions and hosts.

## Conclusion

We analyzed the regional, time and population trends of HFRS, along with the genetic features of HVs from different regions. Our findings provide a theoretical basis for the prevention, control, and vaccine development for HV.

## Data Availability Statement

The original contributions presented in the study are included in the article/**Supplementary Material**, further inquiries can be directed to the corresponding authors.

## Author Contributions

QW, MY, CZ, LA, DH, BZ, ZY, XY, FL conducted the literature research and wrote the paper. XY, LA, BZ, QW, FL, WH, WT, prepared the figures and tables. All authors provided critical review and revisions. All authors contributed to the article and approved the submitted version.

## Funding

This study was supported by the Development Fund of Key Laboratory of Virology Institute of Wuhan University (2020KF003), National Natural Science Foundation of China (81473036, 81171609, U1602223).

## Conflict of Interest

The authors declare that the research was conducted in the absence of any commercial or financial relationships that could be construed as a potential conflict of interest.
